# Parents’ Engagement, Fundamental Movement Skills, and Physical Activity in Early Childhood: A Narrative Review

**DOI:** 10.3390/healthcare14193192

**Published:** 2026-09-28

**Authors:** Ana Alvarez, Stephanie Zinai Gonzalez, Tao Zhang

**Affiliations:** 1 Department of Kinesiology, Health Promotion and Recreation, University of North Texas, Denton, TX 76203, USA; stephaniegonzalez5@my.unt.edu; 2 Baylor College of Medicine, Children’s Nutrition Research Center, Houston, TX 77030, USA

**Keywords:** parent engagement, early childhood, physical activity, fundamental movement skills

## Abstract

**Highlights:**

**What are the main findings?**

- We identified 28 studies that investigated parental engagement and its role in children’s FMSs and PA outcomes, and most were conducted in North America.
- Overall, the evidence is mixed regarding the associations between parents’ engagement and children’s FMSs and PA.

**What are the implications of the main findings?**

- Future FMS and PA research should examine the role of parental influences in children’s FMSs and prioritize fathers, father figures, and single mothers.
- Caregivers, parents, and professionals who work with young children can encourage children to learn FMSs and be physically active.

**Abstract:**

**Background**: Previous research has demonstrated the positive impact of parents’ engagement in young children’s cognitive and social and emotional development. Parents’ engagement in young children’s lives can also be beneficial for the development of healthy behaviors that may reduce childhood obesity. **Objective**: The major purpose of this narrative review was to identify studies that examined the role of parents’ engagement in young children’s fundamental movement skills (FMSs) and physical activity (PA). The primary research question was, what is the role of parents’ engagement in young children’s FMSs and PA outcomes? **Method**: The databases SPORTDiscus, Professional Development Collection, SocINDEX, and Family and Society Studies Worldwide were searched in January 2024 and again in September 2026, and a grand total of 4223 citations were found. Twenty-eight articles were evaluated and underwent data extraction. **Results**: Studies were conducted in North America (10), followed by Europe (8), Australia (3), Asia (3), South America (3), and Africa (1). Parent-engagement variables were evaluated and classified into the following categories: parents’ own PA, parents’ PA practices, parents’ engagement/involvement, and parents’ psychological characteristics. Children’s FMS outcomes included gross, fine, locomotor, object, and overall motor skills. Children’s PA outcomes included time, duration, and percentage of light, moderate, vigorous, and moderate-to-vigorous PA. **Conclusions**: Components of parental engagement are positively associated with children’s FMSs and PA outcomes. Future studies should consider including father, father figures, and single mothers and identifying strategies to include their participation.

## 1. Introduction

Over the past few decades, obesity among young children (i.e., ages 2–19 years) has steadily increased in the United States [1]. According to the World Health Organization [2], 39 million children under age five were classified as having overweight or obese body composition in 2020. Once thought to be conditions that affected people in high-income countries, these conditions are increasing in low- and middle-income countries too [2]. Being overweight or obese during early childhood (i.e., 0–7 years) [3] can have numerous consequences for children, such as cognitive impairment [4] physical ailments [5], and psychological distress [6]. Additionally, some researchers believe that childhood obesity can predict obesity during adulthood [7]. Obesity during adulthood can diminish quality and outlook on life and put adults at increased risk for chronic diseases [8] and mental health issues [9]. Indeed, obesity can have detrimental effects during both childhood and adulthood. From an economic perspective, overweight and obesity impacts healthcare costs and productivity [10]. Therefore, there is a need to investigate this global health epidemic and implement innovative strategies that target and reduce children’s unhealthy behaviors.

### 1.1. Children’s Fundamental Movement Skills (FMSs) and Physical Activity (PA)

Human movements are generally categorized into gross and fine movements. Gross movements incorporate the large muscles of the body, such as the quadriceps (legs), and fine movements can be achieved with smaller muscles, such as the abductor pollicis brevis (hands). Both types of movements are necessary because they work together to move the body [3]. One mechanism that can alleviate overweight and obesity throughout the lifespan is the acquisition of fundamental movement skills (FMSs) during childhood. FMSs are considered to be foundational to more complex movement sequences. They usually include stationary or balance skills, locomotor skills, and object-control or -manipulation skills [11,12,13]. Stationary skills include balancing on one leg at a time or standing on toes only. Locomotor skills are walking, running, or galloping. Object-control skills include catching a ball with both hands and throwing a ball with one hand [13,14]. According to the conceptual model proposed by Stodden and colleagues [15], acquisition of FMSs in early childhood can set a child on a positive spiral of engagement, which includes greater involvement in PA and can indicate a healthier weight. In contrast, when FMSs are not learned early on, children can embark on a negative spiral of disengagement, which includes lower PA and unhealthy and obese weight statuses. PA is any bodily movement that results in energy expenditure [16]. The Physical Activity Guidelines for Americans recommend that preschool-aged children (ages 3–5 years), should aim for engaging in activities of various intensities for three hours daily, and caregivers and parents should encourage children to be active. School-aged children (ages 6–17 years) should aim for 60 min of activity. For youngsters, engaging in regular participation in PA can improve bone health and weight status [17]. Studies have shown the benefits of PA on children’s cognitive (e.g., executive functions and language) [18] and social and emotional development (e.g., social maturity) [19].

Longitudinal studies provide evidence about early-childhood FMSs acquisition and PA in late childhood [20] and adolescence [21]. Barnett et al. [21] found that object-control skills were a significant predictor of adolescent moderate-to-vigorous PA (MVPA), but not locomotor skills. Moreover, other studies have examined PA levels and sport participation of older children who were classified as having low, average, and high FMSs in early childhood [20,22]. These studies showed that early FMSs classifications are significantly associated with later PA. Children of low FMSs decreased in PA over a four-year period, in contrast to children of high PA, whose PA remained stable over time [20]. Furthermore, high-FMSs children demonstrated greater physical strength (e.g., sit-ups and standing broad jumping) and club-level sport participation than children of low FMSs [22].

Both FMSs and PA are necessary for young children’s healthy development. Many early-childhood education centers have designated times for children to be active and develop their movement skills. Researchers have implemented FMSs and PA programs in various early-childhood settings such as day care and Head Start [23,24]. These programs focused on delivering activity sessions during the center’s hours. Additionally, many programs have been delivered by teachers or research staff. However, studies that examine the role of parents’ engagement in young children’s FMSs and PA are rare [25]. Therefore, including parents in structured methods of delivering FMSs and PA content may be a viable way of promoting children’s FMSs and PA development, and perhaps remedying childhood overweight and obesity.

### 1.2. Parents’ Engagement in Young Children’s Development

Parents’ engagement is defined as when a parent or caregiver is present and active in their child’s life [26,27]. Parents are a child’s first teachers and role models and can teach children skills such as identifying letters and numbers [28] and encourage children to be physically active [29]. The term “parent engagement” can be synonymous with parent involvement. Goodall and Montgomery [27] distinguish between these terms by suggesting that parents’ involvement with their children’s school is different from parent engagement in their children’s learning. Researchers have examined various levels of parent engagement and parent involvement and their effect on children’s cognitive and social and emotional development. A systematic review [30] categorized parent involvement as a positive parenting behavior, and, overall, positive parenting behaviors were associated with young children’s executive functions. Other researchers [31] evaluated interventions that were either school-based (i.e., parents’ involvement with the child’s school) or home-based (i.e., parents’ engagement with their child at home). Researchers concluded that parent engagement interventions had a positive effect on young children’s social and emotional competence and that parent engagement interventions were more effective than parent involvement interventions [31].

Parental engagement/involvement has also been evaluated for children’s PA and includes mechanisms such as parents’ own levels of PA, parenting behaviors such as parenting practices, and parents’ attitudes and beliefs toward PA [32]. Recently, Stevenson et al. [33] and Flynn et al. [34] reviewed motor-skill interventions that included direct or indirect parent engagement. These previous reviews evaluated parents’ role in young children’s FMSs or PA, but not both FMSs and PA. Given that previous reviews have not investigated both FMSs and PA, the major aim of this narrative review is to identify and synthesize the evidence on this understudied topic. A narrative review was selected because our research question was broad and exploratory, and our goal was to synthesize the current research on parental engagement, and children’s FMSs and PA [35]. The primary research question was, what is the role of parents’ engagement in young children’s FMSs and/or PA? It was hypothesized that parents’ engagement would have a positive impact on young children’s FMSs and/or PA.

## 2. Materials and Methods

**Figure 1:**
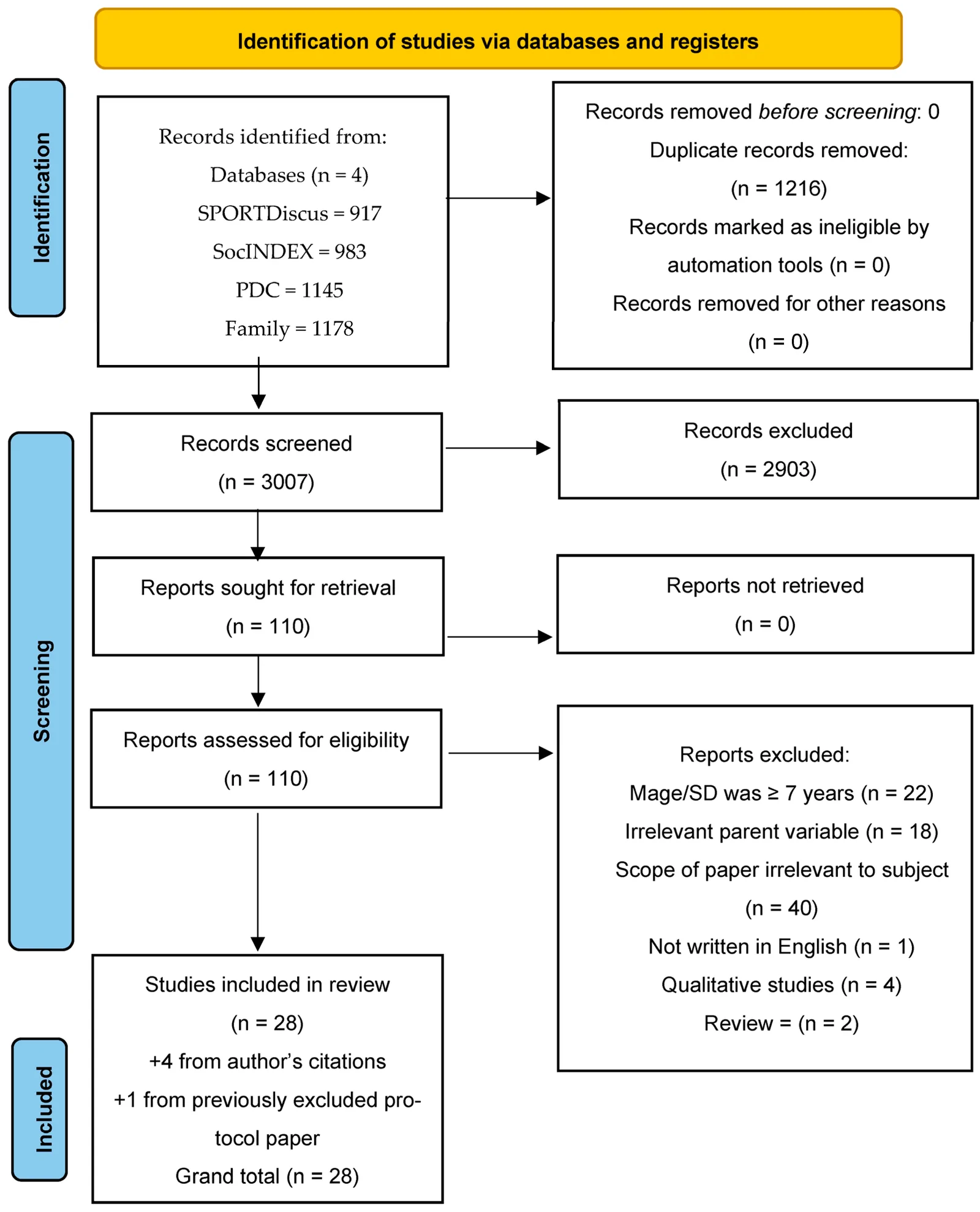
PRISMA flow diagram with results for search.

In preparation for the systematic search, we consulted with our institution’s librarian to identify relevant databases for our topic and keywords. The following databases were searched in January 2024 by author 1: SPORTDiscus, Professional Development Collection, SocINDEX, and Family and Society Studies Worldwide. The keywords and Boolean operators were entered in each of the databases as follows: “parent engagement” OR “parent involvement” AND “motor skills” OR “motor development” AND “physical activity” OR “exercise” AND “young children” OR “early childhood”. Our searches were limited to works published between January 2010 and 31 December 2023, peer-reviewed, written in English, and included access to the full text. Author 1 identified a total of 1975 citations and downloaded them to the Zotero 6.0 reference manager. Using Google sheets, author 1 removed 690 duplicates, leaving 1285 unique citations to review (see Figure 1).

Authors 1 and 2 screened titles and abstracts to determine eligibility for a full review. Citations were eligible for full-text review if they included a parent construct in the title or abstract, a child construct or variable related to FMSs or PA, and the study was about children. Citations were excluded if they were a position or protocol paper, the study focused on sports participation, the study included adolescents/adults, or if the sample was non-typically developing (e.g., spine deficiencies, cerebral palsy, autism, brain dysfunctions, and developmental coordination disorder) (see Table 1).

This reduced the number of citations to 94. Data extraction was guided by Harris and colleagues’ [36] guidelines and included year of publication, country where the study was conducted, primary focus of study, participant characteristics and setting, research design, description of parents’ variable(s), description of children’s variable(s), data source(s), analysis, and outcome(s). These items were selected based on the relevance to the current study. Articles were excluded at this phase if the mean or standard deviation of the children’s age was over 7 years or if the age was not mentioned (N = 22), the parent variable measured was not relevant to the study such as parent education level or demographic characteristics (N = 18), if the overall scope of the paper was not related to the subject (N = 39), or if the paper was not originally written in English (N = 1). This process resulted in 14 studies. Four additional articles were added from the first author’s own citation manager, and one article was added as a follow-up study from a previously excluded citation (i.e., protocol paper). A total of 19 articles were included.

In September 2026, author 1 conducted an updated search, and 9 articles that met the inclusion criteria were identified using a similar screening procedure. For the updated search, the AI companion was incorporated in the original databases to identify articles, and Rayyan [37] was used to deduplicate and screen the articles. In the updated search, four qualitative studies and two reviews were identified. However, they were excluded from this review to focus on quantitative studies. After the updated search, a grand total of 28 articles were included.

## 3. Results

### 3.1. Study Characteristics

Twenty-eight articles met the inclusion criteria (see Table 2 and Table 3). Most studies were conducted in North America (10), followed by Europe (8), Australia (3), Asia (3), South America (3), and Africa (1). Twenty studies were cross-sectional, three were longitudinal, three were randomized controlled trials, one was a quasi-experimental design, and one was a between-subjects comparative design. Two articles from Europe were cross-sectional studies about the Skilled Kids program [38,39]. Across these studies, 71 parent variables were evaluated and classified into the following categories: parents’ own PA, parents’ PA practices, parents’ engagement/involvement, and parents’ psychological characteristics (see Table 4). Fifty-nine variables that measured children’s FMSs (N = 43) and PA (N = 16) were identified (see Table 5).

### 3.2. Children’s Measurement Tools

Twenty-one studies measured children’s FMSs [38,39,40,41,42,43,44,45,46,47,48,49,50,51,52,53,54,55,56,57,58]. Most studies measured children’s FMSs using the Test of Gross Motor Development-3 (N = 6) and the Test of Gross Motor Development-2 (N = 4). Three studies used the Movement Assessment Battery for Children-2 and two studies used the Ages and Stages Questionnaire-3 to assess motor skills. Other tests included the Motoriktest for Vier-bis Sechsjahrige Kinder 4-6, the Peabody Developmental Motor Scales-2, the locomotor skills section of the TGMD-3, and the Progressive Aerobic Cardiovascular Endurance Run (PACER) to measure locomotor skills, measuring functional motor skills (e.g., holding a pencil and catching) by asking parents about their children’s skills; the Motorische Basiskompetenzen im Kindergarten (MOBAK) test; the Bruininks–Oseretsky Test of Motor Proficiency (BOT); six items that were not part of a formal assessment; and one study that used a single-item evaluation of holding a front kick for five seconds to evaluate balance.

Children’s PA was measured with parent questionnaires (three studies) and a checklist (one study, Burdette Outdoor Playtime Checklist). Seven studies used ActiGraph accelerometers (six studies) and Movband4 (one study).

### 3.3. Parents’ Measurement Tools

Parents’ engagement and involvement variables were measured with subjective tools such as questionnaires (e.g., Godin Leisure Time Exercise Questionnaire), survey (i.e., Active Australia Survey), or mobile applications. Three studies measured parents’ PA with ActiGraph accelerometer devices.

### 3.4. Themes of Parents’ Engagement

#### 3.4.1. Parents’ Own PA and Children’s FMSs and PA

Fourteen studies examined the relationship between parents’ own PA and children’s FMSs [38,40,41,42,47,48,56] or PA [41,47,59,60,61,62,63,64,65]. One study reported positive associations between parents’ PA and children’s FMSs [41], two studies reported no relationships [42,47], and Cools and colleagues [40] reported both positive and negative associations, with fathers’ FMSs positively associated with boys’ FMSs. Interestingly, Vukelja et al. [56] assessed both mothers’ and fathers’ PA and averaged their PA score to examine their associations with children’s FMSs scores. Only fathers’ PA was significantly associated with children’s FMSs outcomes. When examining parents’ PA and children’s PA, Carson et al. [60] and Neshteruk et al. [62] reported similar patterns, such that parents who reported low PA also had children who were 2.77 times more likely to have low PA, and parents who spent the most time in MVPA also had children who spent a substantial amount in MVPA over a 10 h period (i.e., 59 min). Two studies [59,61] reported no significant relationships between parents’ PA and children’s PA. Furthermore, Barkin et al. [65] and Ruiz et al. [63] reported positive and negative associations based on PA intensity and duration. Low and moderate PA levels were related, but not vigorous levels of PA. As parents’ duration in MVPA increased from 1 to 10 min and 30–40 min, children’s time in MVPA increased. However, once parents reached 40–50 min in MVPA, children’s time decreased. Finally, mothers who engaged in any leisure-time PA in a four-year period had children with high PA [64]. Overall, the associations between parents’ PA and children’s FMSs and PA, respectively, were mixed and primarily consisted of cross-sectional studies.

**Table 2:** Background (country, focus, participants/setting, and research design) for each study, N = 28.

Author(s) and Year	Country	Primary Focus of Study	Participants and Setting	Research Design
Cools et al. (2011) [40]	Belgium	The focus of this study was to examine the relationships between preschool children’s FMSs, family (parents), and neighborhood contexts.	846 preschool children Mage 5.1 years (*SD* = 0.6), N = 471 boys, N = 375 girls. Setting: Preschool.	Cross-sectional
Honda Barros et al. (2012) [59]	Brazil	The focus of this study was to examine various factors and their relationship to preschool children’s low PA.	265 children, girls’ Mage = 5.90 (*SD* = 0.90), boys Mage = 4.80 (*SD* = 0.80); 220 parents. Setting: Private schools.	Cross-sectional
Carson et al. (2015) [60]	Canada	The focus of this study was to examine the relationships between parents’ and young children’s PA and screen-time behaviors.	738 children, 17.3% of children were 0–24 months, 82.7% of children were 25–71 months, 53.7% were male, 46.3% were female; 738 parents, 91.9% female, 8.1% male. Setting: Parents were recruited from childcare centers and public health and community programs.	Cross-sectional
Salaj et al. (2016) [41]	Croatia	The focus of this study was to examine the motor skills of preschool children enrolled in organized exercise programs and children not enrolled in programs.	78 children, Mage 5.30 years (*SD* = 1.14). Setting not described.	Cross-sectional
Laukkanen et al. (2018) [38]	Finland	The focus of this study was to examine the relationships between physical-activity parenting (PAP) practices and child, family, and environment correlates.	993 children, Mage 5.14 years (*SD* = 1.14). 993 parents’ Mage 35.8 years (*SD* = 5.29), mothers’ N = 865, fathers’ N = 128. Setting: Children attended childcare centers.	Cross-sectional
Szeszulski et al. (2021) [42]	United States	The focus of this study was to explore individual, parent, and home environment correlates of preschooler’s locomotor skills.	144 children, Mage 53.22 months (*SD* = 4.46), 78.9% Hispanic, N = 73 boys, N = 71 girls. In total, 144 parents, Mage 31.72 years (*SD* = 7.74), 93.1% females, 76% Hispanic. Setting: Children attended urban, community-based childcare, and education centers.	Cross-sectional
Sääkslahti and Niemistö (2021) [39]	Finland	The focus of this study was to examine motor development of 2–7-year-old children and their outdoor activities.	1136 children aged 2–7-years-old). Setting: Children attended early-childhood centers.	Cross-sectional
Trost and Brooks (2021) [43]	Australia	The focus of this study was to evaluate the Moovosity program and its impact on parents’ support, children’s FMSs, and children’s PA.	34 parent-and-child dyads, children Mage = 5.30 years (*SD* = 1.3) placed in a control group (N = 17, 47.1% girls) or intervention group (N = 17, 52.9% girls). Setting: Families were recruited from the university’s listserv, database, and word of mouth.	Randomized controlled trial
Rutkauskaitė et al. (2021) [61]	Lithuania	The focus of this study was to identify preschool children’s PA and evaluate its relationship with parents’ PA and parents’ physical fitness.	59 children ages 5–6 years. Total of 101 parents, N = 57 mothers, N = 44 fathers. Setting: Families were from two kindergartens.	Cross-sectional
Nurulfa et al. (2021) [44]	Indonesia	The focus of this study was to examine young children’s FMSs and parents’ level of involvement during the COVID-19 pandemic.	109 children ages 2–7 years (N = 42 children were 2–3 years, N = 36 children were 4–5 years, N = 31 children were 6–7 years). Setting: Parents and children did activities at home.	Cross-sectional
Williams et al. (2017) [45]	Australia	The focus of this study is to explain supported playgroup participation of Australian Aboriginal and Torres Strait Islander families and examine how participation is related to children’s, parents’, and community’s outcomes.	622 children ages 20–53 months, N = 304 girls, N = 304 boys. Total of 554 children identified as Aboriginal, N = 36 were Torres Strait Islander, N = 32 were both. Total of 622 parents, N = 561 mothers, N = 9 mothers, N = 32 aunts/uncles and adoptive/foster parents, N = 20 grandparents. N = 450 parents were Aboriginal, N = 31 were Torres Strait Islander, N = 22 were both, N = 118 were neither. Setting: Families living in urban regional and remote sites.	Longitudinal with 3 waves; Wave 2 children Mage = 25 months (*SD* = 5), Wave 3 Mages = 37 months (*SD* = 5), Wave 4 children Mage = 48 months (*SD* = 5).
Hu et al. (2022) [46]	China	The focus of this study was to examine Chinese preschoolers’ physical fitness, motor development, and family activities.	284 children ages 4–5 years, N = 147 boys, N = 137 girls. Setting: Preschools.	Cross-sectional
Neshteruk et al. (2020) [62]	United States	The focus of this study was to describe parents’ screen time and PA parenting practices and examine differences in children’s PA based on the parents’ behaviors.	319 parent–child dyads, children Mage 3.5 years (*SD* = 0.8), 51% male, 49% female. Total of 319 parents, Mage = 35.4 (*SD* = 6.0), 40% Black, 52% White 6% Hispanic/Latino, 8% Other.	Cross-sectional
Ruiz et al. (2011) [63]	United States	The focus of this study was to examine the relationship between Hispanic parents’ and children’s PA levels.	106 parent–child dyads, children Mage 4.2 years (*SD* = 0.9), 50.9% were females. Parents’ Mage 31.4 years (*SD* = 5.5), 97.2% were females. Total of 81.1% of parents were of Mexican origin, 5.7% were from United States, 13.2% were from another country. Setting: Local community center.	Cross-sectional
Barnett et al. (2013) [47]	Australia	The focus of this study is to examine child, family, and environmental correlates of young children’s FMSs.	76 children and their parents, Mage 4.10 years (*SD* = 0.68), 55% of children were girls. Parents were 95.8% female, 66.2% were born in Australia, 33.8% were born in other countries. Setting: Preschool and childcare centers.	Cross-sectional
Barkin et al. (2016) [65]	United States	The focus of this study was to examine the relationships between parents’ and children’s accelerometer-based PA.	1003 parent-and-child dyads, children Mage 3.9 years (*SD* = 0.9), 75% were Latino, >10% were African American. Setting: Nashville, TN, USA; St. Paul, Minnesota, USA.	Cross-sectional
Brian et al. (2022) [48]	United States	The focus of this study was to examine the role of parental engagement in a motor skill intervention on preschool children’s FMSs and PA.	104 children Mage 48.30 months (*SD* = 6.90), N = 53 boys, N = 51 girls, 74% White, 12.5% Black, 11.5% Hispanic, 1.9% Multiracial. Total of 134 parents, N = 106 mothers, N = 20 fathers, N = 8 guardians. Parents were 71.9% White, 17.8% Black, 8.1% Hispanic, 0.7% multiracial. Setting: Rural, early-childhood center.	Quasi-experimental
Solís-Cordero et al. (2023) [49]	Brazil	The focus of this study was to evaluate the effectiveness of the BEM (Brincar Ensina a Mudar, Play Teaches Change) on children’s development and interactions between caregiver and child.	129 children and their female caregivers, children Mage = 16.3 months (*SD* = 3.6 mos) placed in a control group (N = 63, 54% boys) or Mage 16.1 (*SD* = 3.2 mos) placed in an intervention group (N = 66, 56.1% boys).	Randomized controlled trial
Onyango et al. (2023) [50]	Kenya, Zambia	The focus of this study was to examine the associations between maternal stimulation and child developmental outcomes.	220 mother–infant pairs from Kenya (51.8% female child), mother’s Mage = 26.7 (*SD* = 8.7), 340 mother–infant pairs from Zambia (50.0% female child), mother’s Mage = 27.4 (*SD* = 8.3). In Kenya, mothers were recruited from Ayucha, Border 1, and Wanganga sub-locations of the Awasi Ward. In Zambia, mothers were recruited from the Mwantaya and Chamuku Wards.	Cross-sectional
James et al. (2025) [51]	Canada	The focus of this study was to examine the relationship between parents’ PA support and children’s motor skills and MVPA.	589 children Mage = 4.39 years, (SD = 0.59), 42.4% girls, 57.6% boys, 81.3% White, 18.7% Other. Total of 588 parents, Mage = 35.7 years (SD = 4.2), N = 525 mothers, N = 60 fathers, N = 3 non-biological parents, 85.1% White, 15.9% Other. Setting: Various community organizations.	Cross-sectional
Tornquist et al. (2025) [64]	Brazil	The focus of this study was to examine the relationships between mothers’ and children’s PA.	2974 children at age 1, 51.1% male, 47.9% female, 2645 at age 2, 51.5% male, 48.5% female, 2955 at age 4, 50.5% male, 49.5% female. Mothers who reported any LTPA at age 1 N = 2918, age 2 N = 2466, age 4 N = 2738; any LTPA walking age 1, N = 2858, age 2 N = 2460, age 4 N = 2738, any other LTPA (excluding walking) age 1 N = 2918, age 2 2466, age 4 N = 2700. Setting: Hospital, homes, Research Center.	Longitudinal
Li et al. (2025) [52]	Canada	The focus of this study was to examine individual and environmental correlates (parental) and children’s FMSs.	122 children Mage = 51.1 months (SD = 10.2), 47.1% male, 52.9% female, 68.9% White, 31.1% Other. Total of 122 parents, N = 122 who reported modeling, N = 122 who reported children’s motivation, N = 121 who reported children’s confidence, N = 121 who reported children’s enjoyment, N = 121 who reported motivation/enjoyment/confidence. Setting: Recruited families through online paid social media advertisements.	Cross-sectional
Staiano et al. (2025) [53]	United States	The focus of this study was to examine intervention implementation and parental engagement in the PLAY trial and its association with children’s FMSs.	72 children Mage 4.0 years (SD = 0.8), 57% girls, 63% White, 26% African American. Setting: Childcare centers.	Randomized controlled trial
Samawi et al. (2025) [54]	Indonesia	The focus of this study is to examine social development, parental involvement, and motor development.	600 children ages 48–60 months (53.17%), 61–72 months (46.83%), 48.67% male, 51.33% female. Total of 600 mothers, 35.5% (21–30 years), 52% (31–40 years),12.5% (>40 years). Setting: Kindergartens.	Cross-sectional
Porri et al. (2025) [55]	Italy	The focus of this study was to examine parents’ perceptions of their children’s FMSs.	102 children Mage = 4.41 years (SD = 0.49), 47.1% male, 52.9% female. 92 mothers, 5.2% (20–25 years), 16.7% (25–30 years), 28.1% (30–35 years), 26.0% (35–40 years), 22.9% (>40 years). Setting: Preschool.	Cross-sectional
Vukelja et al. (2025) [56]	Croatia	The focus of this study was to examine the association between parents’ PA and children’s FMSs.	1212 children Mage = 5.2 years. Total of 2287 parents (1173 mothers, 1114 fathers). Settings: State kindergartens.	Cross-sectional
Loh et al. (2024) [57]	Canada	The focus of this study was to evaluate the TEACH courses’ impact on children’s FMSs and parents’ perceptions.	145 children, Mage 42.47 months (SD = 5.96) intervention group, Mage = 39.88 months (SD = 5.77) control group. In total, 50% males and females in intervention group, 45.2% males, 54.8% females in control group. In total, 5.6% White, 5.6% Black, 6.9% Latin-America, 6.9% Asian, 11.2% Arab, 1.4% Multiracial, 1.4% Other from intervention group. In total, 5.5% White, 5.5% Black, 1.4% Latin-American, 4.1% Asian, 11.0% Arab, 0% Other. Setting: Early-childhood centers, childcare centers.	Longitudinal
Konarivand et al. (2025) [58]	France	The focus of this study was to compare the Traditional Taekwondo with TaekFunDo effect on parental engagement and children’s FMSs.	20–22 children ages 4–7 years. Setting: Classroom	Between-subjects comparative design

**Table 3:** Parent and child variables, data sources, analyses, and outcomes for each study, N = 28.

Author(s) and Year	Parent Variable(s)	Child Variable(s)	Data Source(s)	Analysis	Outcome(s)
Cools et al. (2011) [40]	Parents’ (mothers and fathers) PA, parents’ (mothers and fathers) involvement in children’s play, parents’ (mothers and fathers) inquiry about child’s motor development, and parents’ beliefs about children’s PA	FMSs (fine, gross motor skills)	Parent questionnaire (parents’ PA, parents’ involvement in children’s play, parents’ inquiry about motor development, parents’ beliefs about children’s PA); child assessment, Motoriktest für Vier- bis Sechsjährige Kinder (MOT 4–6)	Partial Pearson correlation, two-way ANCOVAs, and least significant differences (LSDs)	Boys: Fathers’ PA was positively associated with boys’ FMSs performance. Higher parental inquiry about girls’ motor development was significantly negatively related to girls’ FMSs. Fathers’ involvement in boys’ active play was significantly related to boys’ greater FMSs performance. Fathers’ involvement in girls’ dance activities was significantly negatively related to girls’ low FMSs. There was a significantly positive relationship between parents’ beliefs (importance) of children’s PA and boys’ FMSs. Fathers who were physically active less than one time per month had children with significantly lower FMSs. Fathers who reported daily PA also had children who scored significantly lower on their FMSs.
Honda Barros et al. (2012) [59]	Parents’ and partners’ practice of PA (yes/no)	PA (duration in minutes)	Parent questionnaire administered face to face (parents’, partners’, and children’s PA)	Descriptives, chi-square, and binary logistic regression	28.5% of mothers and 32.6% of fathers practiced PA. Mothers’ and fathers’ practice of PA were not significant predictors for children’s low level of PA.
Carson et al. (2015) [60]	Parents’ PA (frequency)	PA (frequency and duration)	Parent questionnaire (parents’ PA, Godin Leisure Time Exercise Questionnaire; child’s PA, Statistic Canada’s National Longitudinal Study of Children and Youth (NLSCY)	Descriptives, correlations, and logistic regression models	Parents’ PA was significantly positively related to children’s PA. Parents who were categorized in the low-PA quartile were 2.77 times more likely to have a child in the low-PA quartile, compared to parents who were categorized in the high-PA quartile. The family structure (single- and two-parent homes) was a modifier in that parents’ PA in two-parent homes who were categorized in the first and second PA quartiles had a significantly higher chance of having a child in the low-PA quartile. However, in single-parent homes, parents’ PA was not related to children’s low PA.
Salaj et al. (2016) [41]	Parents’ PA (frequency and duration of moderate and strenuous PA, and walking)	FMSs (locomotor, manipulative skills, and gross motor quotient), PA (duration)	Parent questionnaire (parents’ PA, International Physical Activity Questionnaire, child’s PA); child assessment, Test of Gross Motor Development-2	One-way ANOVA and Bonferroni post hoc test	Parents’ strenuous PA was significantly positively associated with children’s FMSs. Parents’ average daily walking was significantly positively associated with children’s FMSs.
Laukkanen et al. (2018) [38]	Parents’ PAP practices (co-participation, direct support, and encouragement), partners’ PAP practices frequency (co-participation, direct support, and encouragement), parents’ perception of partners’ PAP practices, and parents’ and partners’ exercise frequency	FMSs (locomotor and object-control skills)	Parent questionnaire (PAP practices: co-participation, direct support, encouragement, Family Physical Activity Environment Questionnaire, parents’ perception of partners’ PAP practices, and parent’s and partner’s exercise frequency); child assessment, Test of Gross Motor Development-3	Mann–Whitney U tests, descriptives, independent-sample *t*-test, and hierarchical linear mixed models	Fathers had significantly greater PAP practices and direct support for their children’s PA compared to mothers. Boys scored significantly greater on FMSs compared to girls. The parents’ exercise frequency (partially) significantly predicted their PAP practices. Partners’ PAP practices (partially) significantly predicted the parents’ PAP practices.
Szeszulski et al. (2021) [42]	Parents’ PA parenting practices (encouragement, discouragement of PA), parents’ PA	LS	Parent questionnaire (PA parenting practices, Preschooler Physical Activity Parenting Practices instrument, PA, and Stanford Leisure Time-Categorical Item); child assessments, Progressive Aerobic Cardiovascular Endurance Run (PACER), and CHAMPS motor skill protocol (CMSP)	Descriptives, independent *t*-test, chi-square test, Pearson, point-biserial correlation, and stepwise regression	Parents discouraging PA practices (i.e., promotion of screen time) was significantly associated with greater PACER and CMSP scores. Parents discouraging PA practice (i.e., concern for children’s safety) was significantly associated with lower PACER scores. Parents’ own PA level was not significantly related to children’s locomotor skills on the PACER or CMSP.
Sääkslahti and Niemistö (2021) [39]	Parents answered questions about their child’s PA.	FMSs (locomotor, ball skills, and gross motor quotient)	Parent questionnaire (about child PA); child assessment, Test of Gross Motor Development-3	Descriptives, linear mixed-effects model, and hierarchical linear regression models	Parents’ perspective of their children’s FMSs and enjoyment of PA were related to a child’s better FMSs.
Trost and Brooks (2021) [43]	Parents’ support for child PA	FMSs (locomotor and object-control skills), PA	Parent checklists (5-item scale by Trost et al. (2013) and Burdette outdoor playtime checklist); child assessment, Test of Gross Motor Development-2	General linear mixed models and Cohen’s d	The Moovosity program had a significant impact on the intervention group’s object-control skills, but not on their locomotor skills. The effect was large for object-control skills and medium for locomotor skills. The program did not have a significant impact on children’s PA or parents’ support. The impact on children’s PA was trivial and small for parents’ support of children’s PA.
Rutkauskaitė et al. (2021) [61]	Parents’ PA (frequency, duration, and intensity)	PA, ActiGraph accelerometer, questions answered by parent	Parent questionnaire (parents’ PA and child’s PA); child ActiGraph accelerometer device	Descriptives, ANOVA, chi-square test, and Pearson correlation	72.6% of parents were sufficiently active and 27.4% of parents were insufficiently active. Mothers and fathers rated their PA similarly, but fathers rated their physical fitness higher. Mothers with children 6 years of age indicated their children’s PA significantly higher than mothers of children ages 5 years. There were no significant relationships between parents’ and children’s PA.
Nurulfa et al. (2021) [44]	Parent involvement (support and appreciation of each other, and interaction)	FMSs (stationary, locomotor and object manipulation)	Parent and child, video recordings; child assessment, Peabody Developmental Motor Scale-2	Descriptives	Parental involvement (support and appreciation of each other, and interaction) was higher for children ages 4–5 years
Williams et al. (2017) [45]	Parent engagement in home activities at Wave 2 (2–3 years) and Wave 4 (4–5 years)	FMSs at Wave 4, (ages 4–5 years)	Face-to-face interview (parent engagement); child assessments, functional motor skills (5-parent reported items)	Path analysis	The path between playgroup participation at 2–3 years (through parent engagement in home activities at 4 years) and child functional motor skills at 4 years was not significant.
Hu et al. (2022) [46]	Parental Attitude Toward Physical Activities Scale	FMSs (manual dexterity, aiming and catching, and balance)	Parent questionnaire (Parents’ attitude, Parental Attitude Towards Physical Activities Scale); child assessment, Movement Assessment Battery for Children-2	One-way ANOVAs and hierarchical multiple regression analysis	Parental (positive) attitude was not a predictor of children’s motor ability. Parental attitude was a negative predictor of post coins by non-dominant hand in motor test.
Neshteruk et al. (2020) [62]	Parents’ PA and PA practices	PA	Parent questionnaire (PA and parenting practices); ActiGraph accelerometer; child ActiGraph accelerometer	Latent profile analysis	Three groups of parents were identified: Rewarder, Activity Supportive, and Screen-Time Permissive. There were no significant differences in children’s MVPA between the 3 groups. Children in the Rewarder group spent the most time in MVPA, followed by Activity Supportive and Screen-Time children.
Ruiz et al. (2011) [63]	Parents’ PA (low, moderate, and vigorous)	PA (low, moderate, and vigorous)	Parent and child, ActiGraph accelerometer	Pearson correlations and Cohen’s d	Parents’ and children’s low and moderate PA were significantly correlated. Both Parents’ and children’s vigorous PA were not.
Barnett et al. (2013) [47]	Parents’ perception of children’s motor skills, parents’ confidence in supporting children’s PA, parents’ interaction with children’s PA, and parents’ moderate and vigorous PA (minutes)	PA, FMSs (locomotor and object-control skills)	Parent survey (parents’ perception of children’s motor skills, parents’ confidence in supporting children’s PA, parents’ interaction with children’s PA, parents’ moderate and vigorous PA (minutes); child assessment, ActiGraph accelerometer and Test of Gross Motor Development-2	Multiple regression models	Most parents do not agree that their child does not have good motor skills. Parents’ confidence in children’s PA, parents’ interaction with children’s PA, and parents’ moderate and vigorous PA were not significant predictors of children’s locomotor skills. Parents’ interaction with children’s PA, and parents’ moderate and vigorous PA were not significant predictors of children’s object-control skills. Parents’ confidence of children’s PA was a significant predictor of children’s object-control skills.
Barkin et al. (2016) [65]	Parents’ PA	PA	Parent and child, ActiGraph accelerometer	Descriptives and multiple linear regressions	For each 1 min increase in parents’ light PA, children’s light PA increased by 0.06 min. As parents’ MVPA increased from 1 to 10 min, children’s MVPA increased from 95 to 98.5 min. As parents’ MVPA increased from 30 to 40 min, children’s MVPA increased from 103 to 103.5 min. When parents’ MVPA increased from 40 to 50 min, children’s MVPA decreased from 103.5 to 102.5 min.
Brian et al. (2022) [48]	Parent engagement frequency	FMSs (locomotor and ball skills), PA	Parent survey (engagement frequency); child assessment, Test of Gross Motor Development-3, Movband 4	Descriptives, one-way ANCOVA, and linear regression	For each session attended by parents, children’s locomotor skills increased by 1.87 when assessed at retention. For each session attended by parents, children’s object-manipulation skills increased by 1.95 at retention. When parents attended a session, there was a 1455 step increase in children’s PA.
Solís-Cordero et al. (2023) [49]	Quality of caregiver–child interactions (involvement), and caregiver’s engagement in play activities	FMSs (fine and gross motor skills)	Quality of caregiver–child interactions (Coding Interactive Behavior tool), Caregiver’s engagement in play activities (5 min video) child assessment, Ages and Stages Questionnaire-3	Descriptives, *t*-test, Wilcoxon–Mann–Whitney test, chi-square, linear mixed models, general linear mixed models, and Cohen’s d.	The BEM program did not significantly improve the intervention group’s fine and gross motor skills. However, the intervention group showed improvement in their fine and gross motor skills at endline in comparison to the control group. The BEM program did not have a significant effect on the intervention group’s caregiver involvement. However, caregiver involvement slightly improved at endline compared to the control group. Finally, the BEM program did not have a significant effect on caregiver’s engagement in children’s play activities. Engagement increased for the intervention group at endline in comparison to the control group.
Onyango et al. (2023) [50]	Maternal motor-stimulation activities	FMSs (gross and fine motor skills)	Maternal stimulation (interviews); child assessment, Ages and Stages Questionnaire-3	Descriptives, linear regression, multiple linear regression, and effect size	Maternal motor-stimulation activities increased in both countries, from baseline to endline. Maternal stimulation activities had the largest effect size on children’s gross motor skills.
James et al. (2025) [51]	Parent support for child PA (tangible, intangible, and total PA support)	FMSs (total % score), PA (MVPA)	Parent questionnaire (frequency for child PA support); child assessment, Movement Assessment Battery for Children-2, PA ActiGraph accelerometer	Pearson correlation, moderation, and sensitivity analyses	Total parent support for PA was not significantly associated with child motor skills. Child MVPA significantly moderated the relationship between total parent support for PA and child motor skills. Tangible and intangible parent support were not significantly associated with child motor skills. Regression results show that tangible support was significantly associated with child motor skills, but intangible was not. Total parent support for PA was not significantly associated with child MVPA. Child motor skills significantly moderated the relationship between total parent support for PA and child MVPA. Tangible and intangible PA support were not significantly associated with child MVPA. Only tangible support had a significant effect on child MVPA. Tangible PA had a significant effect on child’s motor skills.
Tornquist et al. (2025) [64]	Mothers’ PA (mothers’ any LTPA, mothers’ walking patterns, and mothers’ any other LTPA, except walking)	PA (overall)	Maternal PA questionnaire; child assessment, ActiGraph accelerometer	Descriptives, group-based trajectory modeling, and linear regressions	At age 2, mothers’ leisure walking was positively associated with children’s PA (1.47 mg more per day). Mothers who reported other LTPAs, aside from walking, had children who had 1.94 mg more PA. Group-based trajectory analysis showed that children with high PA trajectory had mothers who reported any LTPA in more than 1 follow-up and mothers who reported any other PA, except for walking, in more than 1 follow-up.
Li et al. (2025) [52]	Parental PA modeling	FMSs (horizontal jump, hop, overhand throw, underhand throw, and balance)	Parent questionnaire (Preschool Physical Literacy Assessment Tool (Pre-PLAy); child assessment, Test of Gross Motor Development-3 (LS, horizontal jump, hop, BS, overhand throw, and underhand throw), Movement assessment Battery for Children-2 (one-leg balance)	Descriptives, linear, and multiple regression	Linear and multiple regression results showed that parental PA modeling was not a significant correlate of children’s FMSs.
Staiano et al. (2025) [53]	Parental engagement (number of videos accessed, time spent in minutes viewing the videos)	FMSs (locomotor, object-control skills, total percentile, and gross motor index)	Parent engagement, internal usage app; child assessment, Test of Gross Motor Development-3	Descriptives and linear mixed-effects models	Children in the motor-skills intervention (parents had low and high engagement) significantly improved their locomotor, ball skills, and total percentile scores at weeks 12 and 24, compared to baseline. These improvements were higher than the free-play group.
Samawi et al. (2025) [54]	Parental engagement	FMSs (fine and gross motor skills)	Parent questionnaire; child assessment, 6 items for fine motor skills and 6 items for gross motor skills	Structural equation modeling–partial least squares	Parental engagement had a significant positive effect on children’s fine and gross motor skills. Children’s social development significantly moderated the relationship between parental engagement and children’s fine motor skills, but not their gross motor skills.
Porri et al. (2025) [55]	Parental perceptions	FMSs (object movement and self-movement)	Parent survey; child assessment, Motorische Basiskompetenzen im Kindergarten (MOBAK) test	Descriptives, *t*-test, and z-test	There was a statistically significant difference between parents’ perceptions of children’s FMSs and children’s locomotor skills.
Vukelja et al. (2025) [56]	Parental PA level (mothers, fathers, and average for both)	FMSs (below average, average, and above average for TGMD-2 and BOT-2)	Parent survey, Single-Item Physical Activity questionnaire; Bruininks–Oseretsky Test of Motor Proficiency-2; Test of Gross Motor Development-2	Multinomial regression	Paternal, maternal, and average PA for both parents were not significantly associated with BOT-2 outcome groups. Paternal PA was significantly associated with TGMD-2 scores.
Loh et al. (2024) [57]	Parental perceptions of children’s FMSs	FMSs (locomotor, object, and total skills)	Parent questionnaire about their perception of their child’s FMSs competence; child assessment, Test of Gross Motor Development-3	Linear mixed-effects models with random effect, factorial design, Cohen’s d, and intention to treat	Parents’ perceptions of children’s locomotor skills significantly increased in the intervention group, but not the control group. Parents’ perceptions of children’s object-control skills increased significantly in both intervention and control groups. In both groups, parent-reported total FMSs significantly increased.
Konarivand et al. (2025) [58]	Parental involvement (WhatsApp participation %, at-home task-completion rate, satisfaction survey)	FMSs (balance)	Parent, WhatsApp, participation rate, at-home task challenge completion rate, satisfaction survey; child assessment evaluation of holding a front kick for 5 s	Independent-samples *t*-test, ANOVA	Parents of the TaekFunDo group reported greater WhatsApp participation %, at-home completion rate, and higher parental satisfaction. Children in the TaekFunDo group had a significant improvement in balance.

**Table 4:** Summary of 71 parents’ variables.

Author(s) and Year	Variables	Parents’ Own PA	Parents’ PA Practices	Parents’ Engagement	Parents’ Psychological Characteristics
Cools et al. (2011) [40]	Parents’ (mothers and fathers) PA, parents’ (mothers and fathers) involvement in children’s play, parents’ (mothers and fathers) inquiry about child’s motor development, and parents’ beliefs about children’s PA	2	2	2	1
Honda Barros et al. (2012) [59]	Parents’ and partners’ practice of PA (yes/no)	2			
Carson et al. (2015) [60]	Parents’ PA (frequency)	1			
Salaj et al. (2016) [41]	Parents’ PA (frequency and duration of moderate and strenuous PA, and walking)	3			
Laukkanen et al. (2018) [38]	Parents’ PAP practices (co-participation, direct support, and encouragement), partners’ PAP practices frequency (co-participation, direct support, and encouragement), parents’ perception of partners’ PAP practices, and parents’ and partners’ exercise frequency	2	6		1
Szeszulski et al. (2021) [42]	Parents’ PA parenting practices (encouragement and discouragement of PA) and parents’ PA	1	6		
Sääkslahti and Niemistö (2021) [39]	Parents answered questions about their child’s PA.				2
Trost and Brooks (2021) [43]	Parents’ support for child PA		1		
Rutkauskaitė et al. (2021) [61]	Parents’ PA (frequency, duration, and intensity)	1			
Nurulfa et al. (2021) [44]	Parent involvement (support and appreciation of each other, and interaction)			2	
Williams et al. (2017) [45]	Parent engagement in home activities at Wave 2 (2–3 years) and Wave 4 (4–5 years)			1	
Hu et al. (2022) [46]	Parental Attitude Toward Physical Activities Scale				1
Neshteruk et al. (2020) [62]	Parents’ PA, PA practices	1	1		
Ruiz et al. (2011) [63]	Parents’ PA (low, moderate, and vigorous)	3			
Barnett et al. (2013) [47]	Parents’ perception of children’s motor skills, parents’ confidence in supporting children’s PA, parents’ interaction with children’s PA, and parents’ moderate and vigorous PA (minutes)	2		1	2
Barkin et al. (2016) [65]	Parents’ PA	2			
Brian et al. (2022) [48]	Parent engagement frequency			1	
Solís-Cordero et al. (2023) [49]	Quality of caregiver–child interactions (involvement) and caregiver’s engagement in play activities			2	
Onyango et al. (2023) [50]	Maternal motor stimulation activities			1	
James et al. (2025) [51]	Parent support for child PA (tangible, intangible, and total PA support)		3		
Tornquist et al. (2025) [64]	Mothers PA (mothers’ any LTPA, mothers’ walking patterns, and mothers’ any other LTPA, except walking)	3			
Li et al. (2025) [52]	Parental PA modeling		1		
Staiano et al. (2025) [53]	Parental engagement (number of videos accessed; and time spent, in minutes, viewing the videos)			2	
Samawi et al. (2025) [54]	Parent engagement (questionnaire)			1	
Porri et al. (2025) [55]	Parental perceptions (survey)				1
Vukelja et al. (2025) [56]	Parental PA level (mothers, fathers, and average for both)	3			
Loh et al. (2025) [57]	Parental perceptions of children’s FMSs				1
Konarivand et al. (2025) [58]	Parental involvement (WhatsApp participation %, at-home task-completion rate, and satisfaction survey)			3	

**Table 5:** Summary of 59 children’s variables.

Author(s) and Year	Variables	FMSs	PA
Cools et al. (2011) [40]	FMSs, Motoriktest für Vier- bis Sechsjährige Kinder (MOT 4–6) total score	1	
Honda Barros et al. (2012) [59]	PA, duration in minutes		1
Carson et al. (2015) [60]	PA, frequency and duration		1
Salaj et al. (2016) [41]	FMSs, gross motor quotient; PA, duration	1	1
Laukkanen et al. (2018) [38]	FMSs, Test of Gross Motor Development-3 total score	1	
Szeszulski et al. (2021) [42]	LS, completed laps on the Progressive Aerobic Cardiovascular Endurance Run, locomotor score on CHAMPS Motor Skills Protocol	2	
Sääkslahti and Niemistö (2021) [39]	FMSs, gross motor quotient	1	
Trost and Brooks (2021) [43]	FMSs, locomotor skills’ raw score, object-control skills’ raw score; and PA, duration (activity index)	2	1
Rutkauskaitė et al. (2021) [61]	PA, total and average time spent in sedentary, light, moderate, vigorous, and MVPA; questions answered by parent		2
Nurulfa et al. (2021) [44]	FMSs, Peabody Developmental Motor Scale-2 score	1	
Williams et al. (2017) [45]	FMSs, functional motor skills (5-parent reported items) at Wave 4, 4–5 years	1	
Hu et al. (2022) [46]	FMSs, Movement Assessment Battery for Chil-dren-total score	1	
Neshteruk et al. (2020) [62]	PA, minutes in MVPA		1
Ruiz et al. (2011) [63]	PA, minutes per day in light, moderate, and vigorous		3
Barnett et al. (2013) [47]	PA, % of time in MVPA; FMSs, locomotor and object-control skills scores	2	1
Barkin et al. (2016) [65]	PA, minutes per day in light, MVPA		2
Brian et al. (2022) [48]	FMSs, locomotor skills’ raw score, ball skills’ raw score; PA, moves	2	1
Solís-Cordero et al. (2023) [49]	FMSs (fine and gross motor skills)	2	
Onyango et al. (2023) [50]	FMSs (fine and gross motor skills)	2	
James et al. (2025) [51]	FMSs (% total score and PA (MVPA))	1	1
Tornquist et al. (2025) [64]	PA (overall)		1
Li et al. (2025) [52]	FMSs (horizontal jump, hop, overhand throw, underhand throw, and balance)	5	
Staiano et al. (2025) [53]	FMSs (locomotor and object-control skills, total percentile, and gross motor index)	4	
Samawi et al. (2025) [54]	FMSs (fine and gross motor skills)	2	
Porri et al. (2025) [55]	FMSs (object movement and self-movement)	2	
Vukelja et al. (2025) [56]	FMSs (below average, average, and above average for Test of Gross Motor Development-2 and Bruininks–Oseretsky Test of Motor Proficiency-2	6	
Loh et al. (2025) [57]	FMSs (locomotor, object, and total skills)	3	
Konarivand et al. (2025) [58]	FMSs (balance)	1	

#### 3.4.2. Parents’ PA Practices and Children’s FMSs and PA

Seven studies examined the role of parents’ PA practices on children’s FMSs [38,40,42,43,51,52] and PA [43,51,62]. PA practices were measured with questionnaires developed for studies (N = 4), the Preschool Physical Activity Parenting Practices Instrument (N = 1), the Preschool Physical Literacy Assessment Tool (Pre-PLAy) (N = 1), and the Family Physical Activity Environment Questionnaire (N = 1) These questionnaires included a variety of behaviors that parents do to impact their children’s FMSs or PA, such as asking the schoolteacher about the children’s motor skills, co-participating in PA with children, providing direct support for children’s PA, encouragement/praise when children participate in PA, providing transportation to do PA, watching children participate in PA, informing children of the health benefits of PA, and discouraging practices such as screen-time promotion and parents’ concern for children’s safety.

Six studies evaluated parents’ PA practices and children’s FMSs [38,40,42,43,51,52] Cools et al. [40] reported a negative association between parents’ inquiry about children’s motor development and girls’ FMSs. Interesting, Szeszulski et al. [42] found that discouraging PA practices, such as promotion of screen time, was associated with greater locomotor skills performance. Concern for children’s safety was related to lower locomotor scores on the PACER test. Laukkanen et al. [38] examined mothers’ and fathers’ PA practices and found that fathers reported significantly higher practices and provided more direct PA support for their children, compared to mothers. James et al. [51] also examined both tangible and intangible parenting support, and tangible parenting support was significantly associated with children’s motor skills. Li et al. (2025) [52] did not find associations between parental modeling and children’s FMSs.

Three studies examined parents’ PA practices and children’s PA [43,51,62]. Trost and Brooks [43] evaluated the Moovosity intervention’s effectiveness on parents’ support and children’s FMSs. The intervention did not have a significant impact on parents’ support for their children’s PA. Neshteruk et al. [62] conducted a latent profile analysis based on parents’ PA practices and created three groups: Rewarder, Activity Supportive, and Screen-Time Permissive. Children in the Rewarder group spent more time in MVPA compared to the other two groups. Lastly, James et al. [51] found that that total parent support for child PA was not significantly associated with child PA. The findings from these studies are mostly mixed and null.

#### 3.4.3. Parents’ Engagement/Involvement and Children’s FMSs and PA

Ten studies examined parents’ engagement or involvement in children’s FMSs [40,44,45,47,48,49,50,53,54,58] and PA [47,48]. Parents’ engagement was measured with questionnaires (N = 3), face-to-face interviews (N = 2), survey (N = 1), or observation (N = 2). In the updated search, two studies used mobile applications to track parent engagement [53,58]. Brian and colleagues [48] reported positive associations between parents’ engagement and children’s FMSs and PA. Both Barnett et al. and Williams et al. [45,47] reported non-significant relationships between parents’ engagement and children’s FMSs and functional motor skills, respectively. Cools et al. [40] reported both positive and negative associations based on children’s gender. Greater father involvement was related to boys’ higher FMSs performance. In contrast, fathers’ involvement with girls’ dance activities was significantly negatively related to girls’ FMSs. Onyango et al. [50] reported increases in mothers’ motor engagement activities from baseline to endline. Mothers’ engagement had the largest effect size on children’s gross motor skills. Nurulfa et al. [44] conducted an observational study of parents’ and children’s interactions. Researchers measured their appreciation of each other and their interactions. When children were divided into three separate age groups (i.e., 3–4, 4–5, and 6–7 years), it was discovered that the parents of children, ages 4–5, engaged with them more than the parents of children in the other two age groups. Solís-Cordero et al. [49] also employed a similar observation method in their RCT. Caregiver engagement increased from baseline to endline in the intervention group, but the increase was non-significant. Two studies [53,54] reported positive effects when parents used mobile apps. Children in the intervention group improved in their locomotor, gross, and total FMSs score, regardless of their parents’ low- or high-engagement levels [53]. Samawi et al. [54] also reported on how parental engagement had a positive effect on both fine and gross motor skills. In the TaekFunDo program, parents were engaged through the app, and children’s balance increased. Parental engagement seems to also have mixed associations with children’s FMSs and PA, with most studies examining associations between parental engagement and children’s FMSs. Overall, several modalities of assessing parents engagement produced mixed or positive results with children’s FMSs and PA. 

#### 3.4.4. Parents’ Psychological Characteristics and Children’s FMSs and PA

Seven studies examined parents’ psychological characteristics and children’s FMSs [38,39,40,46,47,55,57] and PA [47]. Parents’ psychological characteristics included their beliefs, perspectives, attitudes, or confidence regarding their children’s movements. Two studies [39,40] reported positive associations between parents’ beliefs of the importance of children’s PA and children’s FMSs, and parents’ perspective of their children’s FMSs and children’s FMSs.

Hu and colleagues [46] reported a negative relationship between parents’ attitude and children’s functional motor skills. In Porri et al. [55], parents overestimated their children’s FMSs. In Loh et al. [57], parental perceptions of their children’s FMSs increased for participating in the TEACH course.

In one study, a separate analysis was conducted for locomotor and object-control skills [47]. While parents’ confidence in children’s PA was not a significant predictor of children’s locomotor skills, it was a significant predictor of children’s object-control skills.

In Laukkanen and colleagues’ study [38], parents were asked about their perception of their partner’s PA practices (e.g., co-participation in PA with children and encouragement/praise when children participate in PA). Partners’ PA practices were a significant predictor in parents’ PA practices. The findings from the cross-sectional studies are mixed. 

## 4. Discussion

The major purpose of this narrative review was to evaluate quantitative studies and published studies that examined the role of parents’ engagement in young children’s (i.e., ages 0–7 years) FMSs and PA. Twenty-eight articles met the inclusion criteria. Twenty studies were cross-sectional and primarily conducted in North America (N = 20). Twenty-one studies examined parents’ engagement and children’s FMSs, and twelve studies examined parents’ engagement in children’s PA. Seventy-one parent variables were extracted from the studies and placed in four categories of parents’ own PA, parents’ PA practices, parents’ engagement/involvement, and parents’ psychological characteristics. Additionally, 59 variables were extracted and placed into the broad categories of children’s FMSs and PA.

### 4.1. The Role of Parents’ Engagement in Children’s FMSs

Of the twenty-one studies that examined parents’ engagement in children’s FMSs, ten studies reported that parents’ engagement was positively associated with different components of children’s FMSs performance. In two studies, authors conducted separate analysis for mothers’ and fathers’ engagement and children’s FMSs.

Cools et al. [40] showed that fathers’ engagement (i.e., fathers’ own PA and engagement in play) was beneficial to boys’ FMSs. However, when fathers engaged with girls, girls scored lower in FMSs. Additionally, this study reported how fathers who were physically active less than once a month and exercised daily also had children with low FMSs. Several authors have examined the unique role of fathers in children’s development. Fagan and colleagues [66] examined the role of fathers’ and father figures’ engagement (e.g., reading to their child) and children’s language competence. Children whose fathers read to them at home reported greater language competence. A systematic review by Amodia-Bidakowska et al. [67] focused on father–child play and its role in children’s development, and concluded that it can have a beneficial role in children’s social, emotional, and cognitive development. These studies demonstrate the important role fathers may have in other domains of younger children’s development; however, our results showed otherwise for girls. In the current review, most parent participants reported were female. Davison and colleagues [68] reported on the scarcity of father participants in childhood obesity-prevention research and the lack of studies where researchers conduct analyses on father-related variables. A recent review confirmed that men remain underrepresented in behavioral weight-loss trials [69]. Given that the current review included few father participants and previous research highlighted the underrepresentation of fathers, future researchers should actively recruit fathers and involve them in childhood obesity-prevention interventions to further understand their role in children’s FMSs.

Parents’ engagement yielded mixed findings on children’s locomotor and object-control skills. For example, Trost and Brooks [43], and Barnett and colleagues [47] both reported positive associations between parents’ engagement and children’s object-control skills, but not children’s locomotor skills. These differences may be attributed to the suggestion that object-control skills may require greater perceptual resources, in comparison to locomotor skills [70]. Due to the higher demand needed to execute object-control skills, parents may be more inclined to be engaged with their children when learning these skills. In contrast, Brian and colleagues [48] reported increases in both locomotor skills and object-control skills as parents attended monthly motor skill sessions. In Barnett et al.’s [47] study, parents’ engagement was defined under the broad categories of parents’ own PA, engagement, and psychological characteristics, and in a study by Trost and Brooks [43], parents’ engagement was under the parents’ PA practices category. The greater impact of parents’ engagement in children’s locomotor and object-control skills in Brian and colleagues’ study [48] may be attributed to the nature of parent engagement activities. Parents participated in activities such as learning about the importance of FMSs and opportunities to practice teaching FMSs, and were provided resources (e.g., website and bag of equipment) that may have been meaningful strategies to impact young children’s locomotor and object-control skills. This observation is noteworthy given that, worldwide, young children are more proficient in locomotor skills than object-control skills [71] and participation in object-control skills may predict later childhood and adolescent MVPA. Future researchers can consider instructional sessions embedded with behavioral-change strategies to promote parents’ engagement in young children’s locomotor and object-control skills. In the updated search, two studies used mobile applications to evaluate parents’ levels of engagement. The children in both studies also improved their FMSs. Including technology or other mobile applications to monitor and assess parental levels of engagement may be a promising avenue to explore. Additionally, professionals who work with children (e.g., pediatricians and early-childhood educators), should encourage parents and caregivers to be active with their children and engage with them in different activities.

### 4.2. The Role of Parents’ Engagement in Children’s PA

Twelve studies also examined the role of parents’ engagement in children’s PA. Parents’ engagement can also have a positive effect on young children’s PA. Several studies that examined the relationships between parents’ own PA and children’s PA showed that parents’ PA was positively associated with children’s PA [60,62,63,65]. Furthermore, Carson and colleagues [60] showed that this relationship may be dependent on family structure (i.e., single- and two-parent homes). Essentially, parents’ low PA was related to children’s low PA in two-parent homes; however, this relationship was not observed in single-parent homes. In Carson et al.’s [60] study, most parents were female and part of a two-parent home. Parents in two-parent homes may have greater availability to devote time to their children and role model behaviors for them. Single-parent homes are associated with less time and less supervision toward young children [72]. The disparate characteristics in family structure may influence young children’s development. Parents in single-parent homes may spend less time with their children and as a result, associations between parents’ and children’s PA are absent. Furthermore, single parents may be less available to role model healthy behaviors, such as PA participation. Future researchers should consider the challenges faced by single parents in their children’s PA engagement and provide opportunities for them to be active. Developing strategies and programs specifically for single parents with limited time, while supporting the importance of PA may be a viable avenue to promoting PA in single-parent homes.

Studies in this review also reported on the intensities of children’s PA (i.e., low, moderate, vigorous, and MVPA). Two studies by Ruiz et al. [63] and Barkin et al. [65] showed similar patterns. Children’s low PA was associated with parents’ low PA. Ruiz and colleagues [63] showed that parent and children’s moderate PA levels were not associated. In a similar vein, Barkin et al. [65] also reported that as parents PA increased, so did children’s. Children’s MVPA continued to increase as parents went from 1 to 10 min and from 30 to 40 min. However, once parents reached 40–50 min, children’s MVPA decreased. These studies showed that parent and children’s PA are related, however this relationship is dependent on the amount of time that parents spend in PA and the intensity of the PA. These studies suggest that preschoolers and parents do not engage in higher levels of PA (i.e., moderate, moderate-to-vigorous, and vigorous). More intense PA is necessary to achieve greater health benefits and offset health conditions such as obesity and overweight [63]. Both researchers and practitioners can develop activities and programs to help children, parents, and people of all ages learn about PA of various intensities and how to implement them.

### 4.3. Strengths and Limitations

This study has some strengths. It adds to the literature about parents’ engagement in children’s developmental domains (e.g., cognitive, social, and emotional) by broadening the scope to include motor development and PA. Second, this study expands on the previous findings from Mitchell et al. [32] and includes motor competence. FMSs are considered to be the building blocks of later PA, and understanding parents’ role in this domain could be a potential avenue for early intervention to mediate developmental delays. While this study adds to the motor-development and parent-engagement literature, its limitations must be noted. First, four databases were searched, and we did not search the major biomedical and multidisciplinary databases such as (e.g., PubMed, Web of Science, Embase, Scopus, and PsycINFO). Additionally, one of our inclusion criteria was accessibility to the full-text article. These limitations restrict the studies that we found and reviewed. Second, most studies that met the inclusion criteria were conducted in North America (N = 10). These studies shaped the current perspective on parents’ engagement in children’s motor competence and PA. Third, most of the parents in these studies were female, with only a few studies reporting father participants.

## 5. Conclusions

Childhood overweight and obesity continue to be a public health concern in early childhood. When children learn FMSs and engage in regular PA during early childhood, they are more likely to maintain their PA participation in the middle and later childhood years. Therefore, caregivers, parents, and childhood/early-childhood professionals should encourage and facilitate opportunities for children to be physically active.

In summary, this narrative review evaluated 28 studies to understand parent engagement and how it impacts young children’s (ages 0–7) FMSs and PA. Parents’ engagement (i.e., parents’ own PA, parents’ PA practices, parents’ engagement/involvement, and parents’ psychological characteristics) is beneficial for young children’s FMSs and PA development. Future researchers should consider targeting fathers, father figures, and single mothers in their studies and identifying strategies to enhance their participation. These populations may be unexplored avenues that, once explored, could help mediate effects of childhood overweight and obesity.

## Figures and Tables

**Table 1:** Inclusion and exclusion criteria.

Inclusion Criteria	Exclusion Criteria
Included a parent construct construct/variable	Study included a parent construct/variable about parents’ education/demographic characteristic
Included a child construct related to FMSs/PA	Study included constructs/variables that were not FMSs/PA related
Study was about typically developing children ages 0–7 years	Study included non-typically developing sample (i.e., adolescents and adults)
	Position or protocol paper

Note: FMSs, fundamental movement skills; PA, physical activity.

## Data Availability

No new data were created or analyzed in this study. Data sharing is not applicable to this article.

## Abbreviations

The following abbreviations are used in this manuscript:

FMSs	Fundamental movement skills
PA	Physical activity
